# Imaging brain class I histone deacetylase changes in the Lewy body dementias and Parkinson’s disease

**DOI:** 10.21203/rs.3.rs-6647341/v1

**Published:** 2025-08-11

**Authors:** Anna Goodheart, Chi-Hyeon Yoo, Aline Fassini, Tewodros Dagnew, Rong Ye, Robin Striar, Moqing Quan, Anna Rattray, Tyler Meyer, Erin Peterec, Han Lee, Stephanie Fiedler, Jacob Hooker, Hsiao-Ying Wey, Changning Wang, Stephen Gomperts

**Affiliations:** Massachusetts General Hospital; Massachusetts General Hospital; Massachusetts General Hospital; Massachusetts General Hospital; Massachusetts General Hospital; Massachusetts General Hospital; Massachusetts General Hospital; Massachusetts General Hospital; Massachusetts General Hospital; Massachusetts General Hospital; Massachusetts General Hospital; Massachusetts General Hospital; Massachusetts General Hospital; Massachusetts General Hospital; Massachusetts General Hospital; Massachusetts General Hospital

**Keywords:** Histone deacetylase, Martinostat, dementia with Lewy bodies, Lewy body dementia, Parkinson’s disease

## Abstract

**Background:**

Histone deacetylases (HDACs) are epigenetic molecules responsible for regulation of gene transcription. Altered expression of HDACs has been linked to neurodegenerative disease. Here, we used the class I HDAC PET radioligand [^11^C]Martinostat to quantify and map changes in these molecules in the brain in dementia with Lewy bodies (DLB) and Parkinson’s disease (PD). In this cross-sectional study, we acquired brain PET-MR with [^11^C]Martinostat in 14 DLB (median age 70 years (IQR 14), 21% female), 10 PD (median age 70 (8), 20% female) including four with cognitive impairment and six without, and 17 healthy control (HC) participants (median age 62 (14), 47% female). [^11^C]Martinostat uptake was compared amongst groups using whole brain voxel-wise analysis and targeted region of interest (ROI)-based approaches, adjusted for age and sex. Regional expression was also quantified in postmortem brain bank samples.

**Results:**

Compared to HC, [^11^C]Martinostat uptake in DLB was increased in precentral gyrus (ROI *p* = 0.044) and putamen (*p* < 0.001), as well as in cognitive and limbic circuitry including anterior cingulate (*p* = 0.042) and entorhinal cortex (*p* = 0.023). [^11^C]Martinostat uptake in DLB was decreased in inferior parietal cortex *p* < 0.001) compared to HC, consistent with prior observations in Alzheimer’s disease. In PD, [^11^C]Martinostat uptake was also increased in precentral gyrus (*p* = 0.019 in those with normal cognition, *p* = 0.047 in those with impaired cognition), correlating with both disease duration and stage. In cognitively impaired PD, [^11^C]Martinostat uptake was additionally reduced in inferior parietal cortex (*p* = 0.011), similar to DLB. In postmortem DLB tissue, class I HDAC expression was elevated in anterior cingulate cortex (isoform 1 *p* = 0.041, isoform 3 *p* = 0.024) and reduced in inferior parietal cortex (isoform 1 *p* < 0.001).

**Conclusions:**

These findings reveal evidence of elevated class I HDACs in motor cortex in PD and bidirectional changes in their regional expression in the Lewy body dementias.

## Background

Epigenetic regulation of gene expression is a powerful mechanism by which environmental exposure or pathological cascades can impact cellular and systems function in health and in disease.(1) Epigenetic enzymes responsible for post-translational modifications of histone proteins are thought to have altered expression in neurodegenerative diseases and are thought to contribute to disease susceptibility, learning and memory, synaptic plasticity, neuronal health, and inflammation through their role in altering gene transcription.(1–10) Among these, class I histone deacetylases (HDAC isoforms 1–3) are implicated in the pathogenesis and progression of neurodegenerative diseases such as Parkinson’s disease (PD), and these molecules hold potential as therapeutic targets.(9, 11–27)

In postmortem studies of PD, histone acetylation has been found to be altered(28) in several brain regions including primary motor cortex and the substantia nigra.(17, 26, 29) In contrast, little is known about the dynamics of histone remodeling in the related disease dementia with Lewy bodies (DLB). As PD and DLB share the same core neuropathology, neuronal aggregates of misfolded alpha-synuclein known as Lewy bodies, and as alpha-synuclein can bind to histones and inhibit their acetylation,(30) there is reason to anticipate shared changes in class I HDACs in PD and DLB.

The distributions of regional Lewy body pathologic changes in DLB and in PD differ early in their course, and regional differences in epigenetic regulation of gene expression may contribute to the differential regional vulnerability of these diseases. The distinct topographies of Lewy body pathology match the presenting clinical features of DLB and PD: In DLB, early cognitive and neuropsychiatric impairments, with or without motor decline, on the basis of cortical and limbic alpha-synuclein pathology, and frequently in association with some degree of concurrent Alzheimer’s disease (AD) neuropathologic change(31–35); in PD, largely isolated motor manifestations of parkinsonism on the basis of alpha-synuclein pathology and dopamine cell loss in the substantia nigra pars compacta. Even so, many PD patients develop cognitive and neuropsychiatric impairments as late manifestations of disease, and Parkinson’s disease with dementia (PDD) resembles DLB, the major distinguishing feature being that in PDD, the motor parkinsonian symptoms precede cognitive impairment by at least one year.(35) In association with this clinical convergence of PD and DLB over time, progression of PD is associated with spread of Lewy body pathology to limbic and cortical regions, and cortical alpha-synuclein pathology in advanced PD is indistinguishable from DLB.(36) Whether regional epigenetic regulation in PD and DLB converges with disease progression is unknown.

Until recently, evaluation of HDAC density changes was limited to postmortem studies and animal models. However, the development of the selective class I (HDAC isoforms 1, 2, 3) HDAC PET radioligand [^11^C]Martinostat has enabled antemortem studies of HDAC changes in living people.(37–39) In a recent study of AD, the density of class I HDACs assessed with [^11^C]Martinostat correlated with regional postmortem levels.(40) Unexpectedly, class I HDAC expression was reduced in posterior cortices. Class I HDAC expression was found to mediate the effect of beta-amyloid and tau on brain atrophy and cognitive impairment. Building on these observations, here we acquired [^11^C]Martinostat PET imaging with the aim to map and quantify regional expression of class I HDACs in DLB and PD.

## Methods

### Study Design and Aim

This cross-sectional study aimed to explore the levels of class I HDAC expression in the Lewy body diseases: DLB and PD.

### Participants

Participants were recruited from the Massachusetts General Hospital Movement Disorders and Memory Disorders clinics, as well as from the Massachusetts Alzheimer’s Disease Research Center (MADRC) longitudinal cohort. Diagnoses of referred participants were confirmed by a trained movement disorders neurologist based on established diagnostic criteria.(41–44) Inclusion criteria for the DLB group were age ≥ 55 years and a diagnosis of either DLB(43) or mild cognitive impairment with Lewy bodies.(44) Inclusion criteria for the PD group were age ≥ 55 years and a diagnosis of PD(41) with any cognitive status. Exclusion criteria included history of clinically significant structural brain lesions (e.g. parenchymal tumor, large vessel stroke), history of head trauma, psychiatric disease other than treated depression or anxiety, and use of cognition-impairing medications such as anticholinergics that could impact cognitive function. Deidentified healthy control (HC) scans from other studies at our center were supplemented with additional HCs recruited from the MADRC; all HCs were ≥ 55 years of age. Participants underwent physical and neurologic exams by a trained movement disorders physician including the Unified Parkinson’s Disease Rating Scale (UPDRS),(45) Hoehn and Yahr staging,(46) and Montreal Cognitive Assessment (MoCA).(47) Participants were examined in the “on” state (i.e. on their dopaminergic medications) to optimize performance on cognitive tests sensitive to the motor manifestations of parkinsonism and to limit motion during scanning.

### Imaging acquisition and analysis

[^11^C]Martinostat was synthesized on site, as previously described.(37) Thirty minutes after intravenous injection of 5 mCi of tracer, MRI and PET images were acquired simultaneously using a 3T Siemens TIM Trio with BrainPET insert. The multi-echo magnetization prepared rapid gradient echo sequence was used for MR reconstruction, segmentation, and parcellation using Freesurfer version 6.0 (https://surfer.nmr.mgh.harvard.edu, Boston, MA).(48–50) PET data from 60–90 minutes post injection were reconstructed using a 3D ordinary Poisson ordered subset expectation maximization algorithm for prompt coincidences. An MR-based pseudo-CT was generated using statistical parametric mapping (SPM version 8, https://www.fil.ion.ucl.ac.uk/spm/software/spm8/, London, UK) and was used for attenuation correction.(51) Images were motion corrected with FSL’s (https://fsl.fmrib.ox.ac.uk/fsl/fslwiki, Oxford, UK) MCFLIRT tool, co-registered to MRI, partial volume corrected using PETsurfer with a symmetric geometric transfer matrix implemented in FreeSurfer,(52) and registered to a Montreal Neurologic Institute 152 template brain (https://www.mcgill.ca/bic/software/tools-data-analysis/anatomical-mri/atlases, Montreal, QC). Images were then spatially smoothed (full width at half maximum of 8) and concatenated into diagnostic groups. Standardized uptake value ratios (SUVRs) for region of interest (ROI) analyses were calculated by dividing regional standardized uptake value of each brain region, treating whole brain as a pseudo-reference region.

### Neuropathology

All participants imaged in this study are currently living. Previously donated autopsy brains from different individuals were obtained from the MADRC brain bank. At the time of autopsy, brains donated to the brain bank are divided at the midline, with half frozen at −80°C and half fixed in 10% buffered formalin. After 10–14 days of fixation, tissue blocks are processed on a Thermo Scientific Excelsior ES tissue processor (Thermo Fisher Scientific, Waltham, MA) and embedded in paraffin. Brains donated to the MADRC have undergone neuropathological confirmation of diagnosis, with comprehensive assessment of primary and co-pathologies. From this brain bank, five donors of each of the following clinically-diagnosed groups were selected: DLB, PD, and HC. Tissue from the anterior cingulate, inferior parietal, and precentral gyrus regions were used based on imaging results. 5-micron-thick formalin-fixed paraffin-embedded sections were cleared in xylene and brought to PBS through graded ethanol and water. Antigen retrieval was performed by boiling sections in 10mM citric acid (pH = 6) for 30 minutes. After the solution cooled below 30°C, sections were blocked with 5% NGS and 5% BSA for one hour. Sections were then incubated overnight at 4°C with primary antibodies against HDAC1 (1:100; Santa Cruz, SC-81598), HDAC2 (1:200; Abcam, AB124974), and HDAC3 (1:100; Abcam, AB32369). Following several washes in phosphate-buffered saline, sections were incubated in secondary antibody Alexa Fluor 555 (Thermo Fisher, A-21422). After several washes, sections were incubated with anti-HuD directly conjugated to Alexa Fluor 647 (1:50; Santa Cruz, SC-28299) for 1 hour at room temperature, followed by several washes in PBS. Lipofuscin was blocked using TrueBlack (Biotium, Freemont, CA) following the manufacturer’s instructions. Slides were coverslipped with anti-fade mounting media and imaged using an Olympus VS120 microscope. ImageJ (https://imagej.net/ij, NIH) was used to quantify nuclear staining intensity. Forty cells per section were analyzed, and the average signal intensity in cell nuclei was obtained. Analyses were blinded to experimental conditions.

### Statistical analyses

Comparisons of continuous demographic characteristics amongst more than two diagnostic groups were performed with analysis of variance (ANOVA). Comparisons of demographic characteristics between two groups were made using either two-tailed t-tests for normally distributed variables or Wilcoxon rank sum tests for non-normally distributed variables. Chi-square tests were used to compare categorical demographic variables. Missing variables (e.g. missing MoCA score due to a participant declining to participate in MoCA) were excluded from analyses using those variables. Whole brain voxel-wise group comparisons were performed using an ordinary least squares mixed effects model with age and sex as covariates of no interest, using FSL’s FEAT tool, with a cluster-forming threshold of z > 2.3 and a cluster significance of *P* < 0.05. ROIs were selected based on the voxel-wise results combined with knowledge of the regional pathophysiology of DLB and PD. ROI comparisons were performed using a factorial ANOVA with Tukey-Kramer multiple comparisons corrected post-hoc analysis of the interaction of ROI*diagnosis, adjusted for age and sex. Correlations between SUVRs and continuous clinical characteristics were determined using Spearman correlation, adjusted for age. Three participants had interrupted or truncated scans due to individual participant factors. None of these individuals were outliers (1.5x interquartile range) in whole brain SUV values and were therefore included in the voxel-wise analyses. Two of these three participants, both DLB, were determined to be outliers in the ROI models and were excluded from these models due to undue influence (Cook’s distance (D_i_) > 4/n). Comparisons of cell nuclei signal intensity between DLB and HC brains and between PD and HC brains were performed using two-tailed t-tests of log-transformed data. Residuals from models were examined graphically for conformance to the assumptions of normality and homoscedasticity, as appropriate. A *p* value of < 0.05 was considered significant in statistical tests. Statistical analyses were performed using SAS version 9.4 (https://www.sas.com/en_us/software/on-demand-for-academics.html, Cary, NC) unless otherwise stated.

## Results

### Participants

Fourteen individuals with DLB, 10 individuals with PD (six cognitively normal (PD-normal), four cognitively impaired (PD-impaired)), and 17 HC participants underwent [^11^C]Martinostat PET, simultaneous MRI, and clinical evaluation. Demographics and clinical characteristics are shown in Table 1. There was no statistically significant difference in age amongst groups. Consistent with the known male predominance of DLB and PD,(53) there were more males than females in the disease groups.

### Neuroimaging: Class I HDAC expression in DLB

**Figure 1:**
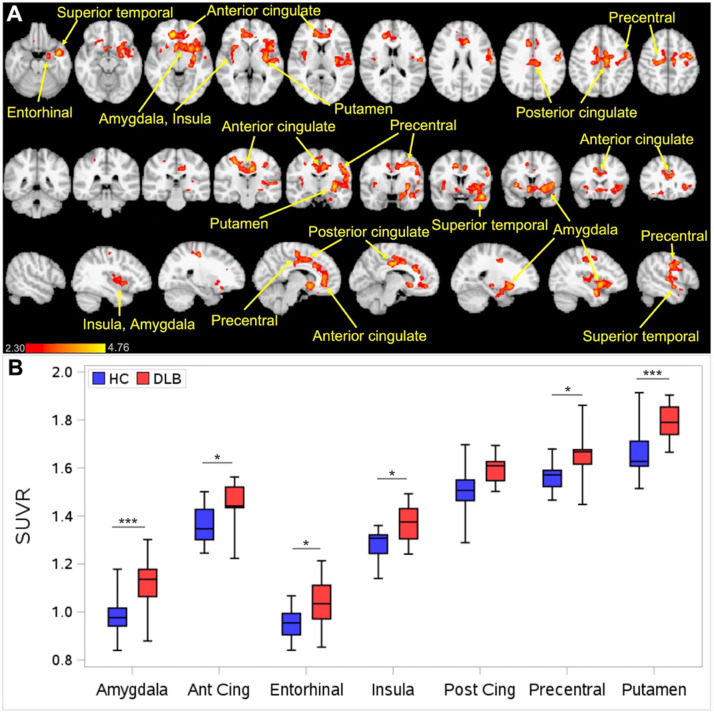
Elevated [^11^C]Martinostat uptake in DLB. **(A)** Voxel-wise analysis showing regions of increased [^11^C]Martinostat uptake in dementia with Lewy bodies (DLB) compared to healthy controls (HC). Scalebar indicates z-score, p_cluster_ < 0.05. **(B)** Region of interest analysis of [^11^C]Martinostat uptake in DLB compared to HC. * *p* < 0.05, *** *p* < 0.001. Box plot displays median value and interquartile range (IQR); whiskers represent 1.5 IQR. SUVR = standardized uptake value ratio.

In voxel-wise contrasts, the topography of increased [^11^C]Martinostat uptake in DLB compared to HC concentrated in motor structures (precentral gyrus (primary motor cortex), putamen) and in components of cognitive and neuropsychiatric circuity including limbic structures (anterior and posterior cingulate, entorhinal cortex, amygdala, and insula) relevant to the clinical characteristics of DLB (Fig. 1A).

To further assess regional increases in [^11^C]Martinostat uptake, ROI-level analyses were pursued in several regions, based on imaging findings and selected for their relevance to the neuroanatomic substrates of DLB neuropathology and its clinical features. [^11^C]Martinostat uptake in DLB was significantly increased compared to HC in motor regions (precentral gyrus, *p* = 0.044; putamen, *p* < 0.001). Similarly, [^11^C]Martinostat uptake in DLB was significantly increased compared to HC in most of the selected limbic and cognitive structures (anterior cingulate *p* = 0.042), amygdala (*p* < 0.001), insula (*p* = 0.036), entorhinal cortex (*p* = 0.023)), with a modest but non-statistically significant elevation in the posterior cingulate (*p* = 0.056) (Fig. 1B).

**Figure 2:**
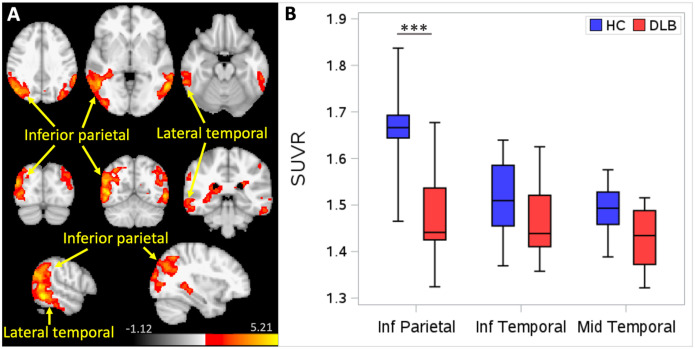
Reduced [^11^C]Martinostat uptake in DLB. **(A)** Voxel-wise analysis showing regions of reduced uptake of [^11^C]Martinostat in dementia with Lewy bodies (DLB) compared to healthy controls (HC). Scalebar indicates z-score, *p*_cluster_ < 0.05. **(B)** Region of interest analysis of reduced [^11^C]Martinostat uptake in DLB compared to HC. *** *p* < 0.001. Box plot displays median value and interquartile range (IQR); whiskers represent 1.5 IQR. SUVR = standardized uptake value ratio.

In contrast to the widespread distribution of increased [^11^C]Martinostat uptake in DLB in regions linked to the disease’s motor and cognitive manifestations, in voxel-wise analyses, [^11^C]Martinostat uptake in DLB was reduced compared to HC in inferior parietal and lateral temporal cortices (specifically inferior and middle temporal gyri) (Fig. 2A). In a follow-up ROI-level analysis of these regions, [^11^C]Martinostat uptake was significantly reduced in the inferior parietal lobe (*p* < 0.001) (Fig. 2B). Regions of increased or decreased [^11^C]Martinostat uptake in DLB were not correlated with disease duration or with clinical measures of motor (UPDRS motor subscale, Hoehn and Yahr stage) or cognitive function (MoCA).

### Neuroimaging: Class I HDAC expression in PD

**Figure 3:**
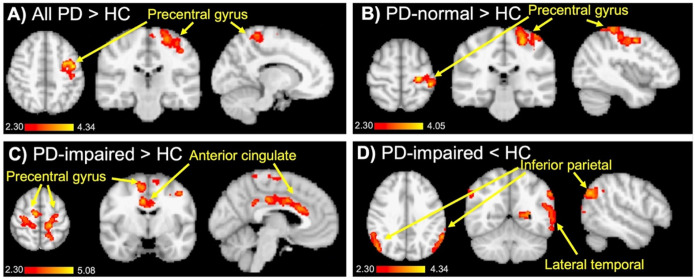
Regional changes in [^11^C]Martinostat uptake in PD. **(A)** Voxel-wise analysis showing elevated [^11^C]Martinostat uptake in Parkinson’s disease (PD) compared to healthy controls (HC). **(B)** Elevated [^11^C]Martinostat uptake in PD with normal cognition (PD-normal) compared to HC. **(C)** Elevated [^11^C]Martinostat uptake in PD with impaired cognition (PD-impaired) compared to HC. **(D)** Reduced [^11^C]Martinostat uptake in PD-impaired compared to HC. Scalebar indicates z-score, p_cluster_ <0.05.

In voxel-wise analyses comparing the PD group to HC, [^11^C]Martinostat uptake was asymmetrically increased in the precentral gyrus (Fig. 3A). No regions of decreased uptake were identified. These results persisted in voxel-wise analyses restricted to the subset of PD participants with normal cognition (PD-normal; Fig. 3B). When the voxel-wise analysis was restricted to the subset of PD participants with impaired cognition (PD-impaired), [^11^C]Martinostat uptake was found to be increased not only in the precentral gyrus but also in the anterior > posterior cingulate cortices (Fig. 3C). In addition, [^11^C]Martinostat uptake in PD-impaired participants was reduced in inferior parietal > lateral temporal cortices (Fig. 3D). These results in the PD-impaired group mirror those observed in DLB.

**Figure 4:**
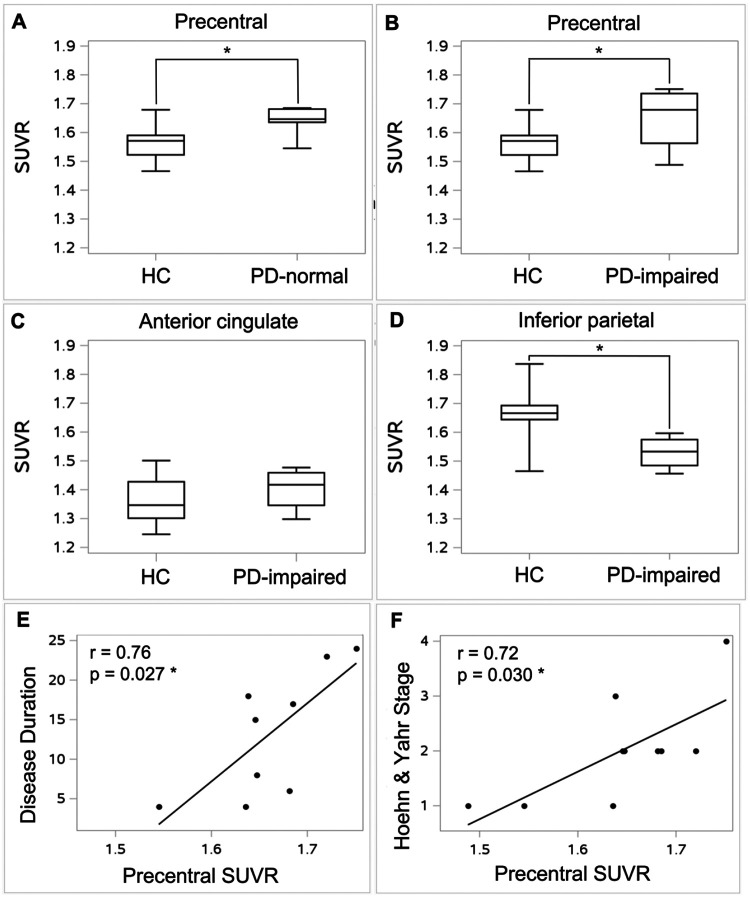
Region of interest analyses of [^11^C]Martinostat uptake in PD. **(A)** Precentral gyrus, Parkinson’s disease with normal cognition (PD-normal) vs healthy controls (HC), **(B)** Precentral gyrus, PD with impaired cognition (PD-impaired) vs HC, **(C)** Anterior cingulate, PD-impaired vs HC, **(D)** Inferior parietal cortex, PD-impaired vs HC, **(E)** Relation between disease duration (years) of all PD participants and precentral gyrus [^11^C]Martinostat standardized uptake value ratio (SUVR), **(F)** Relation of disease stage of all PD participants and precentral gyrus [^11^C]Martinostat SUVR. * *p* < 0.05. Box plots display median value and interquartile range (IQR); whiskers represent 1.5 IQR.

We next pursued ROI-level analyses to further evaluate these findings. [^11^C]Martinostat uptake was significantly increased in the precentral gyrus ROI of both PD-normal (*p* = 0.019) and PD-impaired (*p* = 0.047) subgroups compared to HC (Fig. 4A,B). [^11^C]Martinostat uptake in the anterior cingulate ROI was comparable between PD-impaired and HC (Fig. 4C), despite elevation in anterior cingulate voxels in the voxel-wise analysis. In the PD-impaired subgroup but not the PD-normal subgroup, [^11^C]Martinostat uptake was significantly decreased in the inferior parietal cortex ROI (*p* = 0.011; Fig. 4D). In the PD group as a whole, precentral ROI [^11^C]Martinostat uptake correlated with both disease duration (*p* = 0.027) (Fig. 4E) and Hoehn and Yahr stage of PD (*p* = 0.030; Fig. 4F).

Together, these results show that PD is associated with regional changes in brain class I HDAC levels that overlap with those that arise in DLB. While class I HDAC levels in PD are increased in precentral gyrus, where they vary with duration and stage of disease, cognitive impairment in PD is associated with class I HDAC level increase in anterior cingulate cortex but decrease in inferior parietal cortex.

### Neuropathology: Class I HDAC expression in DLB and PD

To determine whether changes in class I HDAC levels detected with [^11^C]Martinostat in DLB and PD participants were evident in postmortem brain tissue, we evaluated class I HDACs (HDAC1, HDAC2, and HDAC3) in anterior cingulate, inferior parietal cortex, and precentral gyrus tissue samples from DLB and PD patients and healthy controls (HC) without a premortem diagnosis of neurodegenerative disease. Age at death, sex, postmortem interval, and co-pathologies of the autopsy samples are presented in **Additional File 1**. One PD, two DLB, and two HCs had mild AD changes but no more than A3B1C1.(54) Two PD patients had documented cognitive impairment at their last clinic visit.

**Figure 5:**
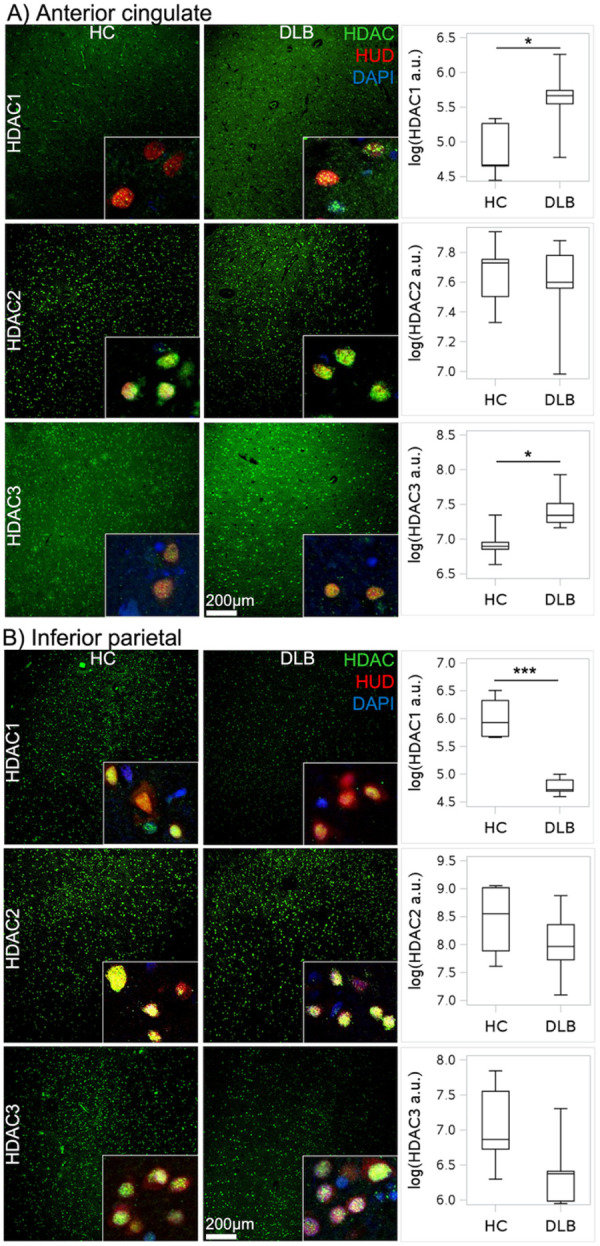
Nuclear intensity counts of histone deacetylase (HDAC) isoforms in human tissue **of (A)** anterior cingulate cortex and **(B)** inferior parietal cortex in dementia with Lewy bodies (DLB) compared to healthy controls (HC) without a diagnosis of neurodegenerative disease prior to death. HUD labels neurons; DAPI labels nuclei. * *p* < 0.05, *** *p* < 0.001. Box plots display median value and interquartile range (IQR); whiskers represent 1.5 IQR.

In DLB anterior cingulate tissue, levels of HDAC1 (*p* = 0.041) and HDAC3 (*p* = 0.024) (but not HDAC2) were increased compared to control tissue. In contrast, in DLB inferior parietal tissue, levels of HDAC1 were reduced compared to control tissue (*p* < 0.001) (Fig. 5). In anterior cingulate or inferior parietal cortex samples from PD and controls, levels of HDACs 1–3 were similar. In addition, in DLB and PD precentral gyrus tissue samples, levels of HDACs 1–3 were comparable to control tissue. Together, these results identify changes in HDAC1 and HDAC3 levels in DLB that may contribute to the regional changes in [^11^C]Martinostat binding observed in anterior cingulate and parietal cortices.

A summary of imaging and neuropathologic findings are presented in Table 2.

## Discussion

Through the use of the novel PET radioligand [^11^C]Martinostat, this study sought to detect DLB and PD associated changes in expression of class I HDACs (isoforms 1, 2, and 3), epigenetic molecules involved in the regulation of gene transcription in response to a cell’s environment or exposures. We observed antemortem changes in class I HDAC expression in disease-relevant brain regions linked to the overlapping clinical manifestations of these diseases. Consistent with the motor impairments of parkinsonism present in both DLB and PD, [^11^C]Martinostat uptake was increased in motor areas in both DLB (precentral gyrus, putamen) and PD (precentral gyrus). In contrast, in DLB but not PD overall, [^11^C]Martinostat uptake was increased in cognitive/limbic areas including the anterior cingulate cortex and was reduced in lateral temporal and inferior parietal cortices. Supporting the shared contribution of these changes in class I HDAC density to cognitive function in DLB and PD, [^11^C]Martinostat uptake in the subset of PD participants with cognitive impairment recapitulated the DLB pattern: increased anterior cingulate uptake and decreased parietal and temporal uptake. Together, these results suggest that DLB and PD, likely as a result of their shared alpha-synuclein neuropathologic changes, share changes in regional class I HDAC density that may contribute to and reflect their overlapping motor and cognitive features.

Consistent with these observations, in postmortem DLB tissue, HDAC1 and HDAC3 levels were elevated in anterior cingulate cortex, and HDAC1 levels were reduced in inferior parietal cortex. A similar neuropathologic pattern was not detected in PD autopsy samples, where only two of the five available autopsy cases had cognitive impairment. Inclusion of tissue samples from the three cognitively normal PD autopsy cases may have contributed to this difference between postmortem and antemortem findings. Despite the increase in [^11^C]Martinostat uptake in the precentral gyrus in DLB and PD and prior demonstration of altered histone acetylation in the precentral gyrus in PD,(29) we did not detect changes in HDAC 1–3 levels in the postmortem DLB or PD precentral gyrus tissue evaluated here. Although the basis for this observation is unclear, tissue sampling may have contributed, as neuropathologic samples were available from only a single hemisphere, while asymmetric motor impairments are frequently seen in these diseases.(35) As the autopsy cases would be expected to be more advanced than the imaged cases, it appears that [^11^C]Martinostat has the ability to detect changes in class I HDAC expression even in early or mid-stage disease and may outperform the autopsy techniques used here in this regard. In any case, the imaging-neuropathological correlations in DLB show that changes in HDAC1 and HDAC3 levels underlie altered [^11^C]Martinostat binding in anterior cingulate and parietal cortex.

Multiple environmental and biological factors regulate HDAC expression, which in turn regulate gene transcription. Although alpha-synuclein pathologic changes can affect HDAC expression,(30) and alpha-synuclein pathology is commonly observed in many of the brain regions demonstrating increased [^11^C]Martinostat uptake in DLB and PD, the changes in class HDAC I density observed here did not reliably coincide with the expected topography of alpha-synuclein pathology. For instance, although [^11^C]Martinostat uptake in DLB and PD was increased in primary motor cortex, consistent with PD postmortem studies demonstrating histone altered HDAC expression in that region,(29) alpha-synuclein pathology in this region is usually only a late manifestation of disease.(34) Thus, not all changes in class HDAC I density that we observed are likely to be a cell autonomous response to alpha-synuclein pathology. Rather, the current results raise the possibility that some changes in class I HDAC density may precede local alpha-synuclein pathology in elements of brain circuits that contribute to the clinical manifestations of these diseases.

Notably, reduced [^11^C]Martinostat uptake in parietal and posterior temporal regions has recently been reported in AD. [^11^C]Martinostat uptake in AD correlated with regional class I HDAC changes postmortem, corresponding with regionally elevated beta-amyloid and tau, and correlated with cortical atrophy and cognitive impairment.(40) As AD co-pathologies of beta-amyloid and tau are common in both DLB and PD dementia,(31–33) comorbid AD pathologic changes may contribute to the decreased [^11^C]Martinostat uptake in parietal and temporal regions observed here in DLB and in cognitively impaired PD participants. The potential contribution of AD co-pathologies to regional changes in class I HDAC density will be important to evaluate in future studies.

Interestingly, [^11^C]Martinostat uptake in the precentral gyrus was significantly correlated with measures of PD clinical progression, including both Hoehn and Yahr stage and duration of disease. Thus, accumulating class I HDAC density, at least in this region, may come with clinical consequences or reflect progression of disease. A diverse set of circuit mechanisms may underlie this observation, including the impact of PD-associated dopamine cell loss on functional connectivity between the striatum and the midbrain and its repercussions on cortical-basal ganglia functional connectivity.(55–57)

The pattern of changes in class I HDAC density in DLB and PD that we observed here has repercussions for clinical trials of class I HDAC modulators targeting these diseases. In cognitively normal PD, our finding of elevated HDAC density in the precentral gyrus fits well with previous observations in PD animal models showing beneficial effects of class I HDAC inhibitors.(11, 14, 16, 18–20, 23) Together, these observations suggest that clinical trials in cognitively normal PD targeting motor function with a class I HDAC inhibitor may have therapeutic potential. In contrast, in DLB or in cognitively impaired PD, the presence of mixed increases and decreases of regional class I HDAC density raises the possibility that exposure to a class I HDAC modulator might have deleterious clinical consequences.

Strengths of this study include the use of [^11^C]Martinostat to image class I HDACs in DLB and PD, a radioligand that binds class I HDACs selectively with low nanomolar affinity(37, 39) and that has been well characterized in the healthy elderly and in several patient populations including AD.(39, 40, 58–60) Another strength is the well-characterized cohort of participants diagnosed according to clinical criteria associated with high diagnostic accuracy, which contributes to generalizability of this sample.(41, 61) The neuropathological assessments provide additional rigor. There are also a number of limitations. Numbers of PD-normal and PD-impaired participants were modest but even so provided imaging results that were consistent with findings in DLB. Another limitation is the cross-sectional design. In this regard, larger, longitudinal studies that account for AD co-pathologies will be of value to determine how changes in regional class I HDAC density relate to the course of these diseases. Limitations of the autopsy data include the limited percentage of brain samples from PD patients with cognitive impairment, single-hemisphere analyses in diseases that have asymmetric motor features, the limited number of autopsy brain samples available which precluded correction for age and sex, and the potential for uncontrolled factors such as mechanism of death and/or postmortem interval to impact measurement of HDAC expression in brain tissue. Although the presence of other co-pathologies including cerebrovascular disease, beta-amyloid, and tau are difficult to avoid in an older population, these co-pathologies were only present at low levels in the brain samples studied.

## Conclusions

Together, these findings demonstrate changes in brain class I HDAC density in DLB and PD in brain regions that contribute to the motor and cognitive features of these diseases. These results suggest that HDAC expression in the synucleinopathies is complex and that development of potential therapies directed at these molecules would necessitate a nuanced approach.

## Supplementary Material

Supplementary Files

This is a list of supplementary files associated with this preprint. Click to download.

- Additionalfile1.docx

## Figures and Tables

**Table 1:** Demographics

A)	HC	DLB	PD
**n**	17	14	10
**Age**	62 (14)	70 (14)	70 (8)
**Sex, % female**	47%	21%	20%
**Disease duration, years**	-	5 (3) *	15 (12) *
**MoCA**	-	20 (9) *	27 (9) *
**UPDRS III**	-	20 (14)	20 (11)
**Hoehn & Yahr stage**	-	2.5 (1) *	2 (1) *
B)	PD-normal	PD-impaired	
**n**	6	4	
**Age**	70 (4)	67 (13)	
**Sex, % female**	33%	0%	
**Disease duration, years**	7 (11) †	23 (6) †	
**MoCA**	29 (3)	20 (13)	
**UPDRS III**	20 (9)	18 (23)	
**Hoehn & Yahr stage**	2 (1)	2.5 (2)	

**(A)** Healthy control (HC), dementia with Lewy bodies (DLB), and Parkinson’s disease (PD) participants. **(B)** The 10 PD participants in (A) stratified by cognitive function. Unless otherwise noted, data are presented as median (interquartile range). MoCA = Montreal Cognitive Assessment (out of 30 points; a lower score indicates worse cognitive performance). UPDRS III = Unified Parkinson’s Disease Rating Scale part III (motor examination, out of 108 points; a higher score indicates worse motor function). Hoehn & Yahr = Hoehn and Yahr stage (up to 5; higher value indicates greater disability).

* *p* < 0.05 difference between DLB and PD.

† *p* < 0.05 difference between PD-normal and PD-impaired.

**Table 2:** Summary table

	DLB	PD-impaired	PD-normal
Motor			
**Precentral gyrus**	**↑**	**↑**	**↑**
**Putamen**	**↑**		
Limbic			
**Amygdala**	**↑**		
**Anterior cingulate**	**↑** *	**↑** ^†^	
**Entorhinal**	**↑**		
**Insula**	**↑**		
**Posterior cingulate**	**↑** ^†^		
Cortical			
**Inferior parietal**	**↓** *	**↓**	
**Inferior temporal**	**↓**		
**Middle temporal**	**↓**		

Summary of regional [^11^C]Martinostat uptake in dementia with Lewy bodies (DLB), Parkinson’s disease with impaired cognition (PD-impaired), and Parkinson’s disease with normal cognition (PD-normal), compared to healthy controls.

* Corroborated by neuropathologic findings.

† Voxel-wise only.

## Data Availability

The datasets used and/or analyzed during the current study are available from the corresponding author on reasonable request.

## Abbreviations

HDAC: histone deacetylase
PET: positron emission tomography
DLB: dementia with Lewy bodies
PD: Parkinson’s disease
MR: magnetic resonance
IQR: interquartile range
HC: healthy control
ROI: region of interest
AD: Alzheimer’s disease
PDD: Parkinson’s disease dementia
MADRC: Massachusetts Alzheimer’s Disease Research Center
UPDRS: Unified Parkinson’s Disease Rating Scale
MoCA: Montreal Cognitive Assessment
CT: computed tomography
SUVR: standardized uptake value ratio
ANOVA: analysis of variance
